# Position Paper of INoEA Working Group on Long-Gap Esophageal Atresia: For Better Care

**DOI:** 10.3389/fped.2017.00063

**Published:** 2017-03-31

**Authors:** David C. van der Zee, Pietro Bagolan, Christophe Faure, Frederic Gottrand, Russell Jennings, Jean-Martin Laberge, Marcela Hernan Martinez Ferro, Benoît Parmentier, Rony Sfeir, Warwick Teague

**Affiliations:** ^1^Department of Pediatric Surgery, UMC Utrecht, Utrecht, Netherlands; ^2^Department of Medical and Surgical Neonatology, Newborn Surgery Unit, Bambino Gesù Children’s Hospital-Research Institute Rome, Rome, Italy; ^3^Department of Pediatrics, Université de Montréal, Montreal, QC, Canada; ^4^Pôle enfant, Hôpital J de Flandre CHRU de Lille, Lille, France; ^5^Department of Pediatric Surgery, Boston Children’s Hospital, Harvard Medical School, Boston, MA, USA; ^6^Montréal Children’s Hospital, McGill University, Montreal, QC, Canada; ^7^Department of Pediatric Surgery, J. P. Garrahan National Children’s Hospital, Buenos Aires, Argentina; ^8^Department of Pediatric Surgery, Robert Debré University Hospital, APHP, Paris, France; ^9^Department of Surgery, Jeanne de Flandre Hospital, Lille, France; ^10^Department of Pediatric Surgery, The Royal Children’s Hospital, Melbourne, VIC, Australia

**Keywords:** long-gap esophageal atresia, definition, diagnosis, management, centers of expertise

## Abstract

INoEA is the International Network of Esophageal Atresia and consists of a broad spectrum of pediatric specialties and patient societies. The working group on long-gap esophageal atresia (LGEA) set out to develop guidelines regarding the definition of LGEA, the best diagnostic and treatment strategies, and highlight the necessity of experience and communication in the management of these challenging patients. Review of the literature and expert discussion concluded that LGEA should be defined as any esophageal atresia (EA) that has no intra-abdominal air, realizing that this defines EA with no distal tracheoesophageal fistula (TEF). LGEA is considerably more complex than EA with distal TEFs and should be referred to a center of expertise. The first choice is to preserve the native esophagus and pursue primary repair, delayed primary anastomosis, or traction/growth techniques to achieve anastomosis. A cervical esophagostomy should be avoided if possible. Only if primary anastomosis is not possible, replacement techniques should be used. Jejunal interposition is proposed as the best option among the major EA centers. In light of the infrequent occurrence of LGEA and the technically demanding techniques involved to achieve esophageal continuity, it is strongly advised to develop regional or national centers of expertise for the management and follow-up of these very complex patients.

INoEA is the International Network of Esophageal Atresia and consists of a broad spectrum of pediatric specialties and patient societies. Esophageal atresia (EA) is not only a congenital malformation that warrants surgical correction, but the malformation is complex, frequently associated with other concomitant anomalies, and requires life-long multidisciplinary follow-up and support.

In the EA spectrum, long-gap esophageal atresia (LGEA) is only a small portion (10%), but the inability to perform a primary esophageal anastomosis poses additional challenges to bring the two esophageal ends together and restore continuity (1, 2).

There are several techniques available, reflecting that no one technique is ideal, and the patients are left with many challenges to overcome (2–5). Also, the infrequent occurrence of LGEA means that few surgeons will develop adequate experience, which will preclude the development of improved techniques. Most surgeons will see less than 1 LGEA every 10 years, so even the most senior surgeons may be very inexperienced with their treatment challenges (6).

A literature search leaves only small retrospective series or case histories with little incentive for technical advances (2–5). What has become clear is that it is best to try to preserve the native esophagus (3). Where the distance between the two ends appears to preclude approximation directly after birth (immediate primary repair), this may become possible after waiting for several weeks (delayed primary repair).

In the literature, it is unclear what exactly defines LGEA. Quite often, a difficult to approximate esophageal atresia (EA) with the distal esophagus ending in the trachea (type C) is determined as a LGEA. Probably, in experienced hands, most of these types of EA could have been brought together with a primary anastomosis (7, 8).

It is, therefore, important to come to a clear and unambiguous definition of LGEA:

Definition

Any esophageal atresia (EA) that has no intra-abdominal air should be considered a long gap and is advised to be referred to a center of expertise (CoE)

and

All other types that technically prove to be difficult to repair are not necessarily long gap, but should be referred to CoEs in any case, after the first failed attempt

A CoE can be defined as:

A CoE is a pediatric surgical center that is equipped and experienced in the treatment of patients with long-gap esophageal atresia (LGEA)

What are the criteria for a CoE?

A CoE:

- Has a protocol describing the management of all types of EA, including LGEA
- Has a highly specialized department of neonatology and anesthesiology available for pre-, peri-, and postoperative care
- Preferably has prenatal diagnosis and counseling facilities
- Routinely performs a preoperative rigid trachea-bronchoscopy
- Has extensive experience in repair of all types of LGEA
- Can manage all kinds of concomitant anomalies associated with LGEA (VACTERL association, laryngeal anomalies)
- Can manage all sequelae, like anastomotic stricture, gastroesophageal reflux disease, tracheomalacia, tracheoesophageal fistula (TEF), and recurrent TEF.
- Has a structured follow-up program including pediatric surgery, neonatology, pediatric pulmonology, pediatric gastroenterology, pediatric radiology, pediatric cardiology, pediatric urology, ENT, orthopedics, genetics, pediatric neurology, psychology, social work, occupational therapy, dietician, speech therapy, and physiotherapy. Provides basic life support
- Organizes transition to adult care
- Develops collaboration with family and patients support groups
- Has/collaborates with a dedicated database
- Has a research program dedicated to EA

After having defined the correct diagnosis, the next issue is how to determine the gap between the ends of the esophagus and the existence of concomitant anomalies.

There was general consensus that a preoperative rigid tracheobronchoscopy (9, 10) is mandatory to exclude the presence of a proximal fistula that has been described to be found in more than half of the cases (11) and to determine if tracheomalacia is present.

In order to be able to perform a contrast study of the distal esophagus a (laparoscopic) gastrostomy may be constructed. Some centers use bougies to determine the distance between the proximal and distal pouch. The preference depends on the center and the experience with their chosen technique.

There was general consensus that a cervical esophagostomy should be avoided, because this may increase the difficulty of a delayed primary anastomosis, or the use of jejunal interposition as such a graft may not be able to reach up to the neck without microvascular supercharging. Good nursing care with the use of a Replogle^®^ sump drain will adequately prevent aspiration from saliva in the proximal pouch (12).

In recent years, the esophageal traction technique has become more popular, and this can even be performed thoracoscopically directly after birth without the need for a gastrostomy (5).

Only if primary esophageal anastomosis is not possible in the judgment of the CoE, esophageal replacement techniques should be used. In major centers for EA, the jejunal interposition is preferred, because the graft grows at a similar rate as the child and maintains intrinsic motility (13). In addition, the risk of gastroesophageal reflux, leading to pulmonary complications in the long term is less than in gastric pull-up and colonic interposition.

The advantage of a gastric pull-up is that blood supply is very good, only one anastomosis is necessary. However, reflux is a dominant issue (14). When only 1–3 cm of defect remains, some formation of a gastric tube can avoid the usage of replacement technique, although this is also not without complications of gastroesophageal reflux (15).

Colon interposition is mainly reserved as a last option, when all other techniques have failed or are considered unfeasible. Sequela include kinking due to inappropriate growth, bulging of the graft in the neck, persistent stasis of food residue in the graft with reflux, and aspiration and gastroesophageal reflux (2, 16).

Centers of expertise should master the whole armamentarium to be able to deal with restoration of the esophagus.

If not possible to preserve the native esophagus, the following options are available

- Jejunal interposition
  - In the neonate with vascularized stalk
  - In the older child with micro anastomosis
- Gastric pull-up
  - In some cases where only 1–3 cm defect remain, it may be possible to perform an alternative technique of tube elongation.
- Colon interposition
  Because there are so many sequelae like kinking etc., this is often a last resort.

This brings us back to what a CoE should be. Is every pediatric surgical center automatically a CoE for LGEA? There are countries where designated centers of expertise have been appointed by the government, like in France or the Netherlands (17). Some countries have major esophageal airway centers, like in the USA (18, 19). Distance should not really be an issue, because the reconstruction usually takes place at a later time and traveling is not exceptional for patients nowadays. Many patients travel all over Europe or even to the USA for restoration of the esophagus in LGEA. It is probably more a matter of acceptance or acknowledgment that patients can travel more freely to centers of expertise.

Regional or national discussions should be started about referral centers for specific conditions.

For anomalies, such as congenital diaphragmatic hernia, there are ECMO centers available, biliary atresia is being concentrated into a limited number of centers in the UK as in many other countries (20). Similar centers could be determined for bladder extrophy, Hirschsprung disease, tracheomalacia, and EA.

Pediatric surgeons are dedicated to give their patients the best care for some specific congenital malformations that requires centers of expertise.

## Author Contributions

This is a position paper by the working group on long-gap esophageal atresia. All authors have equally contributed to this paper.

## Conflict of Interest Statement

The authors declare that the research was conducted in the absence of any commercial or financial relationships that could be construed as a potential conflict of interest. The reviewer FK and handling editor declared their shared affiliation, and the handling editor states that the process nevertheless met the standards of a fair and objective review.
